# Ultrasonographic Evaluation of Labor Patterns: A Prospective Cohort Study in Greece

**DOI:** 10.3390/jcm14155283

**Published:** 2025-07-25

**Authors:** Kyriaki Mitta, Ioannis Tsakiridis, Andriana Virgiliou, Apostolos Mamopoulos, Hristiana Capros, Apostolos Athanasiadis, Themistoklis Dagklis

**Affiliations:** 1 Third Department of Obstetrics and Gynecology, School of Medicine, Faculty of Health Sciences, Aristotle University of Thessaloniki, 54642 Thessaloniki, Greece; kiki93mit@gmail.com (K.M.); anvirgiliou@hotmail.gr (A.V.); amamop@auth.gr (A.M.); apathana@auth.gr (A.A.); dagklis@auth.gr (T.D.); 2 Department of Obstetrics and Gynecology, Nicolae Testemitsanu State University of Medicine and Pharmacy Chisinau, MD-2004 Chișinău, Moldova

**Keywords:** intrapartum ultrasound, labor patterns, angle of progression, head perineum distance

## Abstract

**Background/Objectives:** Recent changes in obstetric practices and population demographics have prompted a re-evaluation of labor patterns. This study aimed to characterize labor patterns in a Greek pregnant population using ultrasound and compare them with established labor curves. **Methods:** A prospective cohort study was conducted at the Third Department of Obstetrics and Gynecology, School of Medicine, Faculty of Health Sciences, Aristotle University of Thessaloniki, Greece, over a two-year period (December 2022 to June 2024). Transabdominal ultrasound was used to determine the fetal head position and transperineal ultrasound was used to measure angle of progression (AoP) and head–perineum distance (HPD) during labor. Maternal and labor characteristics, including body mass index (BMI), parity, labor duration, and mode of delivery, were recorded. Statistical analysis included mixed linear models to assess the relationship between AoP, HPD, and cervical dilatation. **Results:** In total, 500 parturients were included in this study. Women entered the active phase of labor approximately 5 h before delivery, with AoP increasing sharply and HPD decreasing rapidly at this point. Cesarean section (CS) cases showed a slower increase in AoP compared to vaginal deliveries (VDs), with CS cases having a mean AoP of 117.9° (95% CI: 111.6–124.2°) at full dilation, compared to 133.4° (95% CI: 130.6–136.2°) in VD. HPD values declined more slowly in CS cases, with a mean HPD of 45.1 mm (95% CI: 40.6–49.6 mm) at full dilation, compared to 36.4 mm (95% CI: 34.3–38.5 mm) in VD. Epidural analgesia was associated with steeper increases in AoP and decreases in HPD in the final 2.5 h before delivery, while oxytocin administration accelerated these changes in the last 3–4 h. The mean time to delivery was 3.19 h (95% CI: 2.80–3.59 h) when AoP reached 125° and 3.92 h when HPD was 40 mm (95% CI: 3.53–4.30 h). BMI in women who gave birth via CS was significantly higher compared to VD (32.03 vs. 29.94 kg/m^2^, *p*-value: 0.008), and the total duration of labor was shorter in VD compared to CS and operative vaginal delivery (OVD) (8 h vs. 15 h, *p*-value < 0.001 and 8 h vs. 12 h, *p*-value < 0.001, respectively). Birthweight was also lower in VD compared to CS (3103.09 g vs. 3267.88 g, *p*-value: 0.05). **Conclusions:** This study provides the first ultrasonographic characterization of labor patterns in a Greek population, highlighting the utility of ultrasound in objectively assessing labor progression.

## 1. Introduction

In 1955, Friedman et al. described the patterns of fetal head descent during labor and the progression of cervical dilatation, establishing the “labor curve of Friedman” [1]. This labor curve included both the latent and active phase of the first stage of labor, as well as the second stage. During the active phase of the first stage, Friedman described three distinct component phases: an acceleration phase, a phase of maximum slope, and a deceleration phase. These observations were based on clinical examinations and provided the foundations for understanding labor progression.

Current changes in obstetric practices and population demographics have prompted new interest in studying labor progression, with greater emphasis placed on fetal head descent patterns rather than on cervical dilatation alone. In particular, in 2002, Zhang et al. revised the labor curves and identified notable differences from Friedman’s original observations [2]. They reported that the latent phase of the first stage of labor was significantly longer, especially among primiparous women. Furthermore, the mean duration for cervical progression from 4 cm to 10 cm dilatation was longer. Importantly, the transition from the latent to the active phase was observed at 6 cm of cervical dilatation, in contrast to the Friedman’s original threshold of 4 cm.

In more recent years, technological advances have provided new tools for assessing labor progression [3]. Intrapartum, transperineal, and transabdominal ultrasound is a reliable method of assessing fetal head station and descent [4,5]. In 2018, the International Society of Ultrasound in Obstetrics and Gynecology (ISUOG) published guidelines regarding the use of ultrasound in labor as an objective alternative to the traditionally subjective and less accurate clinical methods of assessing fetal head station [6].

The aim of this study was to describe the labor curves in the Greek pregnant population using ultrasound during labor and to compare these findings with the labor curves described by Friedman and Zhang.

## 2. Material and Methods

### 2.1. Study Design

This was a prospective cohort study conducted at the Third Department of Obstetrics and Gynecology, School of Medicine, Faculty of Health Sciences, Aristotle University of Thessaloniki, Greece, during a 2-year period (December 2022 to June 2024). This study was approved by the ethics committee of the Aristotle University of Thessaloniki (14.12.2022/49/2022). Informed consent was provided to all participants and no incentives were provided.

Maternal characteristics, including age, body mass index (BMI), and parity, labor characteristics, including total duration of labor, use of epidural analgesia and oxytocin, whether labor commenced spontaneously or was induced, mode of delivery, i.e., vaginal delivery (VD), operative vaginal delivery (OVD), and cesarean section (CS), and neonatal parameters, i.e., birthweight, were recorded. This prospective observational study included all women who were admitted to the maternity unit either in spontaneous labor or for labor induction, with a cephalic fetal presentation regardless of gestational age or the attending obstetrician. Inclusion was not limited by maternal age, parity, or any specific risk factors. All obstetricians participating in this study had previously completed formal training in intrapartum ultrasound techniques.

To ensure randomization and minimize selection bias, three on-call shifts per month were randomly selected in advance, during which intrapartum ultrasound assessments were systematically performed alongside routine clinical management. On average, the Department conducts approximately 1500 deliveries annually. Of these, roughly 30% are elective cesarean sections, which were excluded from this study, as no labor process occurs in these cases. Abnormal fetal presentation was another exclusion criteria. This sample was collected over 72 on-call days.

Cervical dilatation was examined digitally. For cases requiring labor induction, the Bishop score was assessed to determine the appropriate induction method. A favorable Bishop score (≥6) indicated that amniotomy, with or without oxytocin administration, was the preferred induction method. Conversely, an unfavorable Bishop score (<6) necessitated the use of cervical ripening agents.

Labor induction followed the guidelines set by international organizations [7]. For cervical ripening, prostaglandins were employed: either a 3 mg vaginal suppository of dinoprostone (PGE2) with a minimum safe interval of 6 h before initiating oxytocin, or 25–50 mcg of misoprostol (PGE1) as an initial dose, followed by oxytocin at least 4 h after the last misoprostol dose. If cervical changes were insufficient with minimal uterine activity following the initial dose of dinoprostone or misoprostol, a second dose was administered after 6 h [8].

### 2.2. Sonographic Technique

All parturients were examined with both transabdominal and transperineal ultrasound during labor to determine the fetal head position and fetal head descent, using a Voluson P8 (General Electric, Boston, MA, USA) ultrasound machine. The sonographic evaluation of the fetal head station was conducted using both transabdominal (to assess fetal head position) and transperineal ultrasound in either the midsagittal or axial plane. The ultrasound probe was positioned between the labia majora, at the level of the fourchette, with the patient in a semi-recumbent position [6]. The ultrasound was conducted by the on-call obstetricians; all of them had received appropriate training in intrapartum ultrasound prior to the beginning of this study. The sonographic assessment was initiated upon the introduction of parturients to the labor ward, with serial evaluations conducted as per clinical needs.

**Scheme 1:**
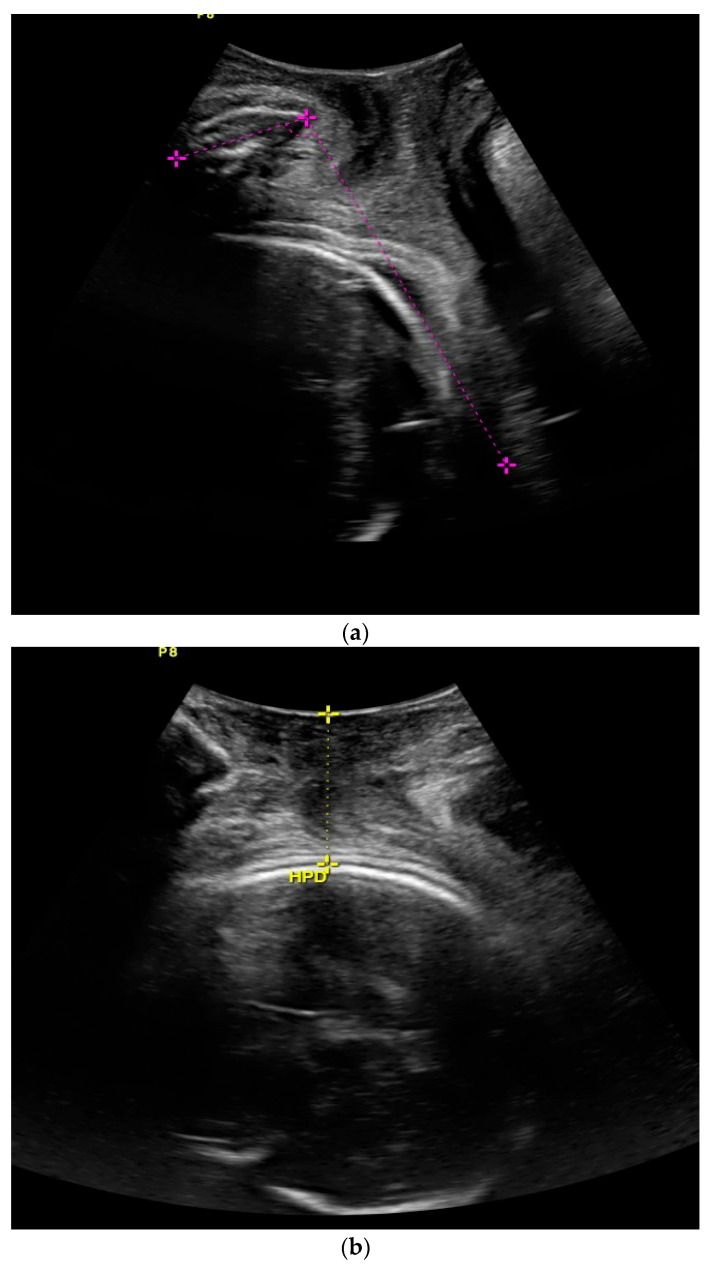
(**a**) (AoP) (The two pink lines form an angle which is the angle of progression), (**b**) (HPD).

The angle of progression (AoP) is defined as the angle between the long axis of the pubic bone and a line drawn from the lowest edge of the pubis tangential to the most inferior bony part of the fetal skull. It is considered a reliable and reproducible parameter for assessing fetal head descent [9,10]. An AoP of 116° has been identified as corresponding to fetal head station 0 (zero) [9] (Scheme 1a).

The head–perineum distance (HPD) is measured by placing the probe between the labia majora, ensuring that the soft tissues are fully compressed against the pubic bone. The transducer is adjusted until the contour of the fetal skull is sharply visualized, indicating that the ultrasound beam is perpendicular to the skull. The HPD is then determined on a frontal transperineal scan as the shortest distance between the outer bony margin of the fetal skull and the perineum [6,11] (Scheme 1b).

### 2.3. Statistical Analysis

Power sample size calculation was conducted to determine the appropriate sample size. Using a 95% confidence level, a statistical margin of error of 5%, and assuming the annual number of pregnant women in the population is around 85,000, the appropriate sample size is calculated to be approximately 384. However, we chose to include slightly more than the minimum required to account for potential dropouts or missing data. Therefore, all individuals who met inclusion criteria and had complete assessment data were included in the analysis. These individuals were selected based on predefined inclusion criteria and completeness of data. No post hoc exclusions or selections were made to manipulate results.

Means, standard deviations (SDs), and medians and interquartile ranges were used to describe the quantitative variables. Absolute (n) and relative (%) frequencies were used to describe categorical and ordinal variables. Sample characteristics were compared among the delivery methods via the Pearson’s chi-square test, Fisher’s exact test, analysis of variance (ANOVA), and Kruskal–Wallis test. Mann–Whitney and Kruskal–Wallis tests were used to associate AoP, HPD, and dilatation values at the first and last examination with sample characteristics, since, via the Kolmogorov–Smirnov criterion, a non-normal distribution was found. To check for type I error, due to multiple comparisons, the Bonferroni correction was used whereby the significance level is 0.05/κ (κ = number of comparisons).

Raw observational data were used for statistical analysis, without applying a predefined labor curve model. The change in AoP, HPD, and dilatation values over time were described by curves which were estimated by means of 4th degree polynomials. The 95% confidence intervals (95% CI) of the estimates were also calculated. These curves were made for the whole sample as well as depending on the type of delivery, whether they were nulliparous or not, the type of onset of labor, and whether the OVD/VD cases with spontaneous labor onset had received an epidural or oxytocin. AoP, HPD, and dilatation values were associated with each other through mixed linear models, from which regression coefficients (β) and their standard errors (SEs) were derived. Through mixed linear models, time to delivery was also estimated according to different AoP and HPD values. All reported *p* values are two-tailed. Statistical significance was set at *p* < 0.05 and analyses were conducted using R and STATA 15.0.

## 3. Results

### 3.1. Study Population

Data from 500 deliveries were collected and analyzed. In total, 1216 examinations were performed in the study population. The mean number of examinations per woman was 2.43 (SD = 1.53), ranging from 1 to 10.

Maternal, pregnancy, and labor characteristics are presented in Table 1, overall and by type of labor. Women’s BMI (*p* = 0.011), total duration of labor (*p* < 0.001), and birthweight (*p* = 0.012) differed significantly by type of delivery. More specifically, following a Bonferroni correction, it was found that the BMI in women who gave birth by cesarean section (CS) was significantly higher compared to VD (*p* = 0.008), the total duration of labor was significantly shorter in VD compared to both OVD (*p* < 0.001) and CS (*p* < 0.001), and the birth weight was significantly lower in VD than in CS (*p* = 0.05). In addition, percentages of nulliparous women differed significantly between delivery types (*p* < 0.001), as did onset of labor (*p* = 0.007) and percentages of oxytocin administration (*p* = 0.05). However, after Bonferroni correction, the percentage of induction of labor was found to be significantly lower in VD compared to CS (*p* = 0.005) and OVD (*p* < 0.001), the percentage of nulliparous women was significantly lower in VD compared to CS (*p* < 0.001), while no significant differences were found in rates of oxytocin administration.

### 3.2. Labor Patterns

**Figure 1:**
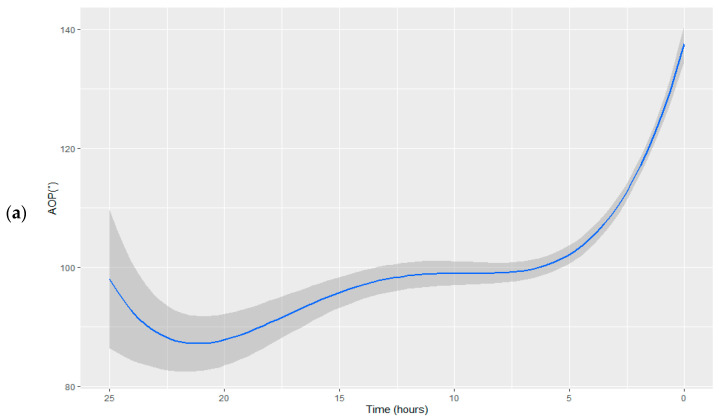

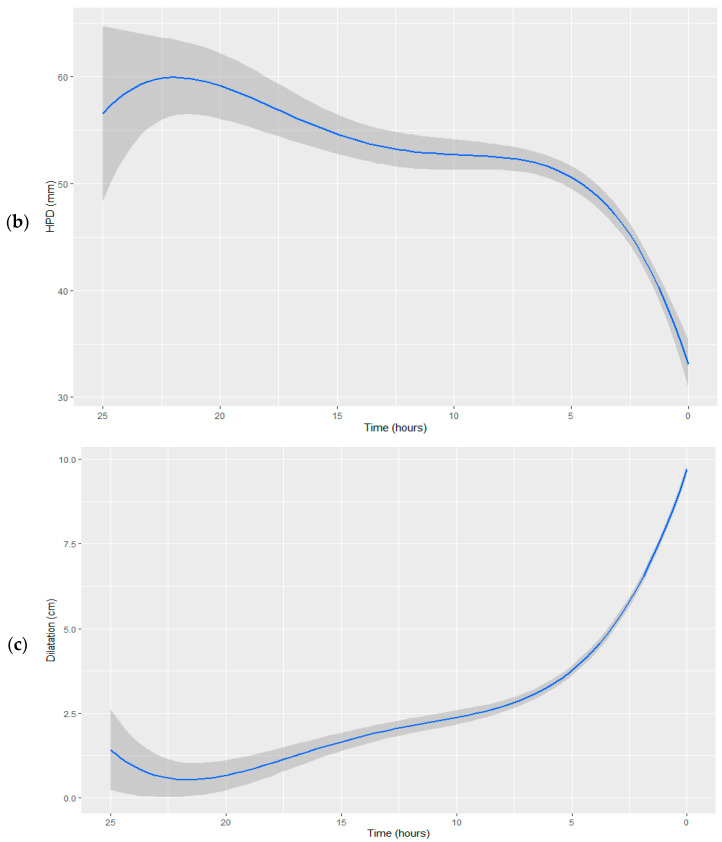
Changes in AoP (**a**), HPD (**b**), and dilatation (**c**) values until delivery in total sample.

The progress in HPD, AoP, and cervical dilatation are presented in Figure 1. The average duration of the active phase (from dilation ≥ 4 cm, as defined by Friedman) to delivery was approximately 5 h. The active phase (dilation ≥ 4 cm, per Friedman) to delivery averaged 5 h. During this period, AoP rose sharply while HPD declined steeply. Before the active phase, AoP remained relatively stable, and HPD decreased gradually until 5 h before delivery, after which the decline accelerated. On average the HPD was 36 mm when the dilatation was close to full (approximately 9 cm). At 2.5 h before delivery, mean values were: AoP 116°, HPD 45 mm, and dilation 7–8 cm.

**Figure 2:**
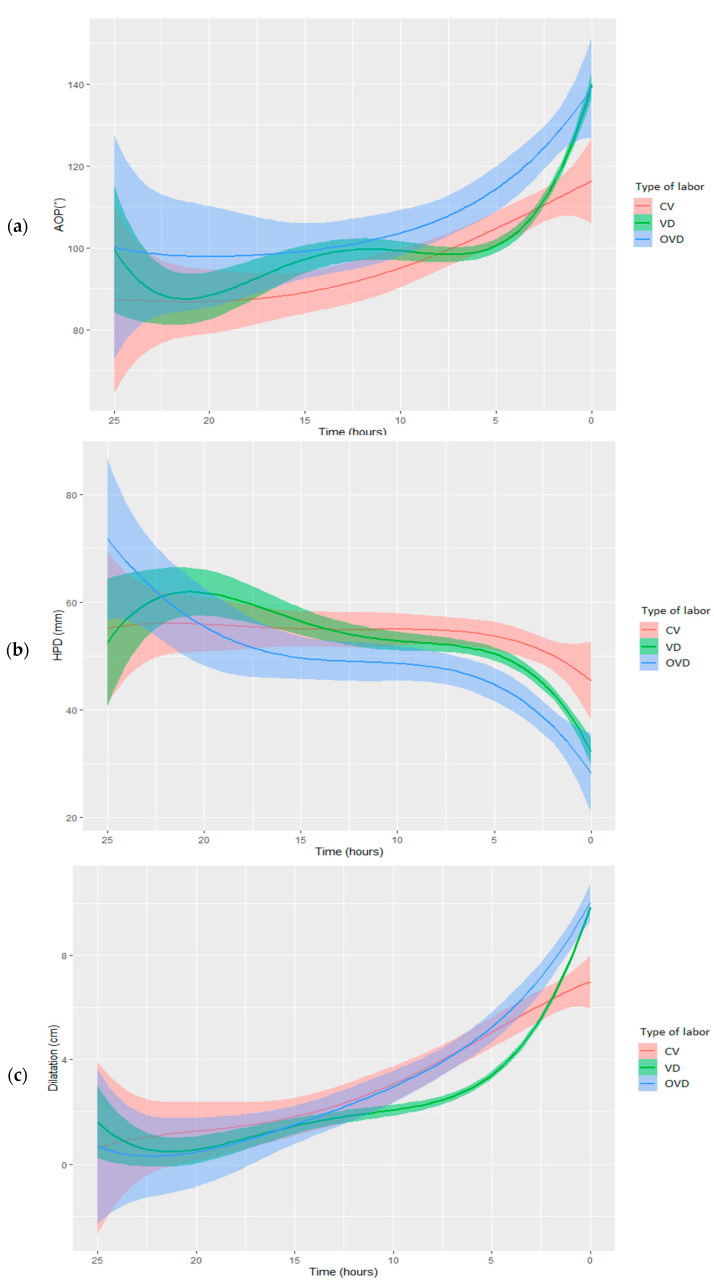
Changes in AoP (**a**), HPD (**b**), and dilatation (**c**) values until delivery, by type of delivery.

The variation and mean change in HPD, AoP, and cervical dilatation from onset of observation to delivery, by type of labor, are presented in Figure 2. In VD, AoP fluctuated until 5 h before delivery, then rose steeply. In OVD and CS, AoP increased steadily but at slower rates, especially in CS. HPD in OVD declined continuously, most sharply after 7.5 h. In CS, HPD remained relatively unchanged. In VD, HPD rose until 20 h before delivery, then declined; first gradually, then more sharply from 5 h before delivery. Cervical dilatation increased in all groups: steadily in OVD, steeply after 5 h in VD, and more gradually in CS.

**Figure 3:**
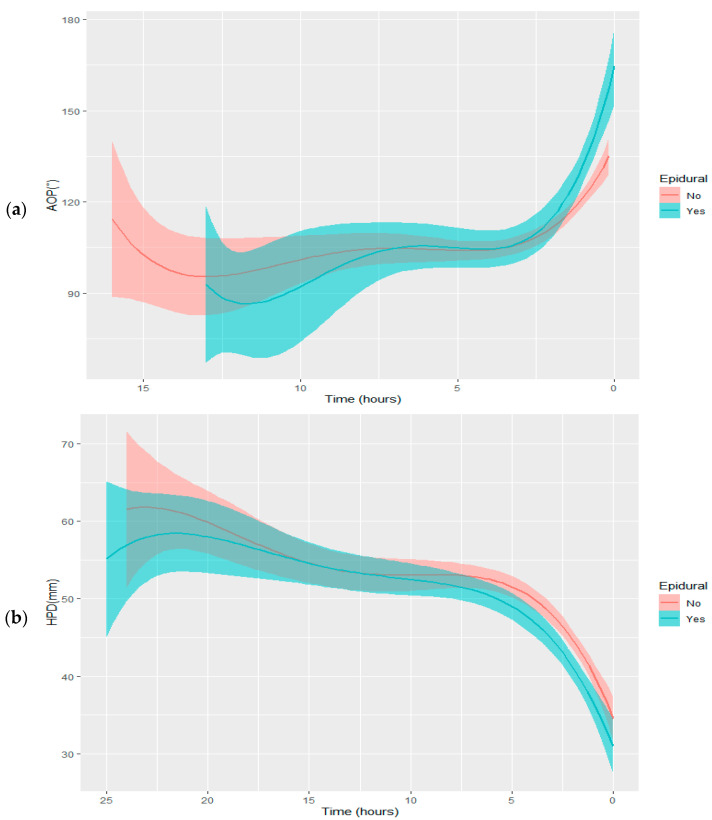

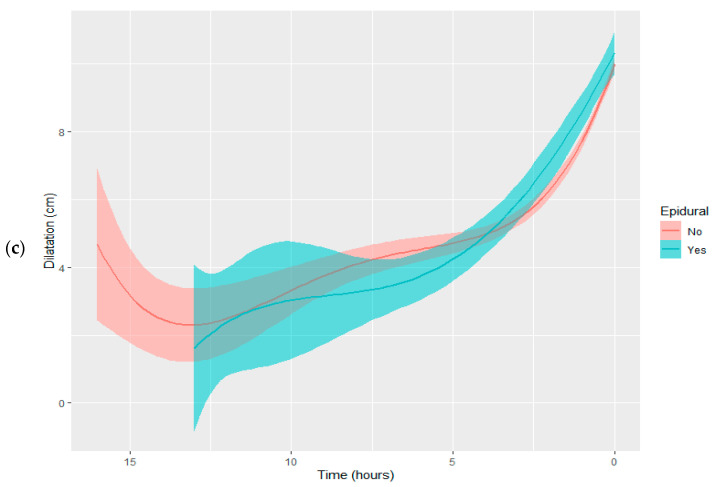
Changes in AoP (**a**), HPD (**b**), and dilatation (**c**) values until delivery of VD or OVD cases with spontaneous onset of labor, associated with administration of epidural or not.

**Figure 4:**
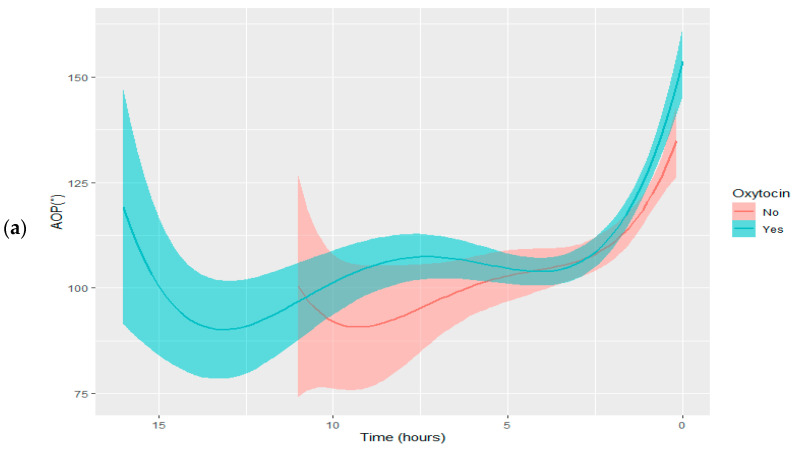

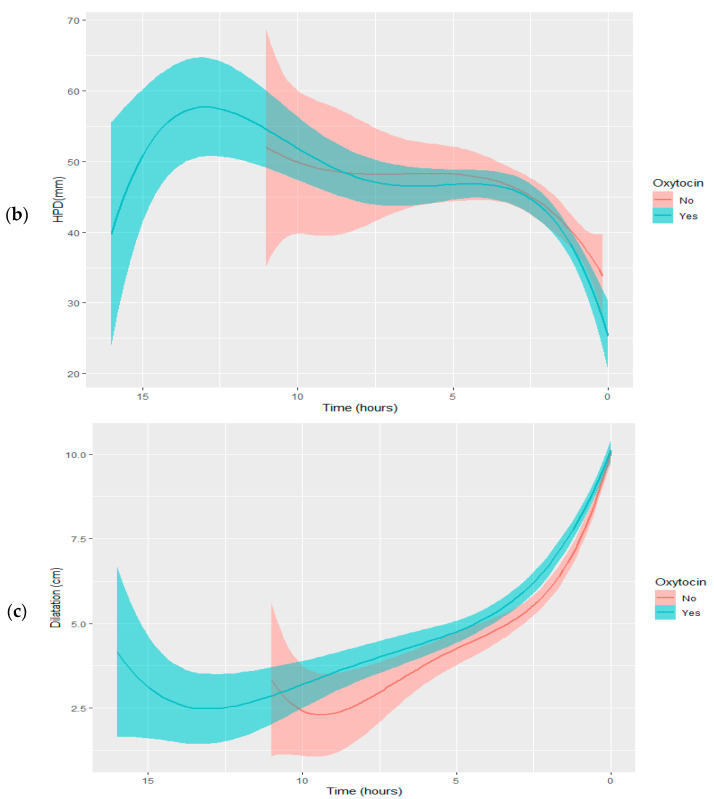
Changes in AoP (**a**), HPD (**b**), and dilatation (**c**) values until delivery of VD or OVD cases with spontaneous onset of labor, associated with administration of oxytocin or not.

In Figure 3 and Figure 4, the changes in AoP, HPD, and dilatation values of VD/OVD cases with spontaneous onset of labor until delivery, associated with administration or not of an epidural and oxytocin, respectively, are presented. Overall trends in AoP, HPD, and dilatation were similar regardless of epidural use. However, the final increase in AoP and dilatation, and the final decrease in HPD, beginning around 2.5 h before delivery, were steeper in women who received an epidural. A similar pattern was observed with oxytocin. While overall changes in AoP, HPD, and dilatation were comparable between those who did and did not receive oxytocin, the final rise in AoP and dilatation, and the final decrease in HPD, starting approximately 3–4 h before delivery, were more pronounced in the oxytocin group.

**Figure 5:**
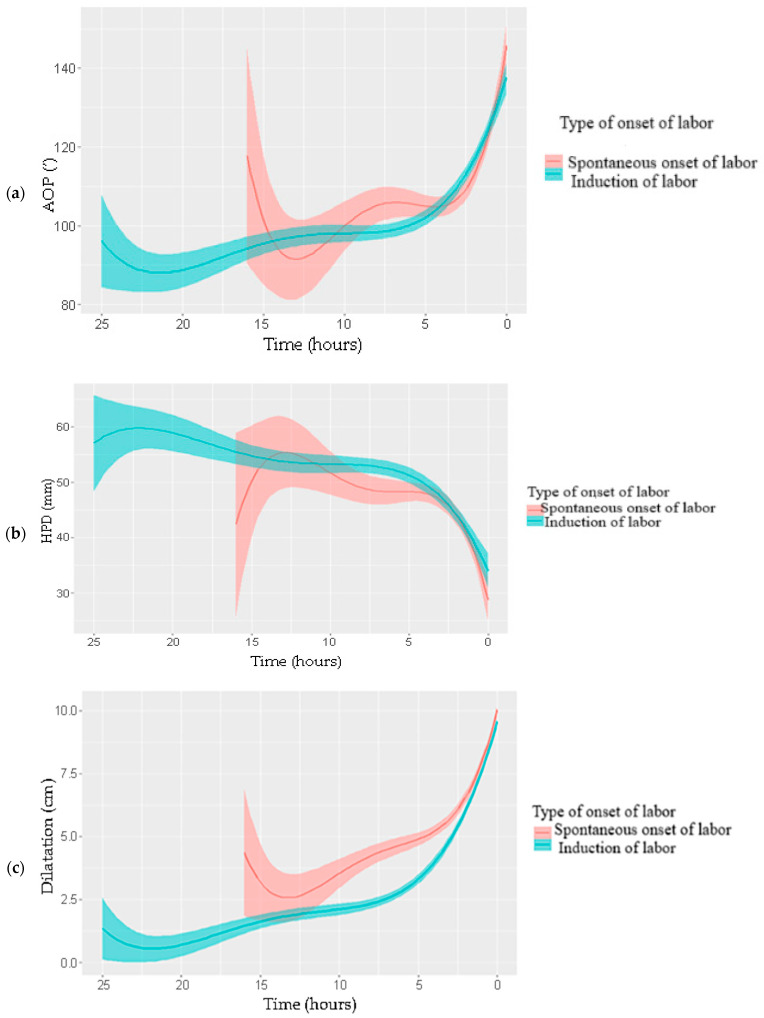
Changes in AoP (**a**), HPD (**b**), and dilatation (**c**) values until delivery by type of onset of labor; spontaneous onset vs. induction of labor.

The changes in AoP (a), HPD (b), and cervical dilatation (c) in parturients, depending on the onset of labor (spontaneous onset of labor vs. induction of labor) are depicted in Figure 5. In induced labor, AoP increased steadily, with a mild slope initially that steepened about 5 h before delivery. In spontaneous labor, the rise in AoP was sharper but occurred over a shorter period, beginning 3–4 h before delivery. Similar patterns were seen in HPD and cervical dilatation: HPD declined and dilation increased more gradually in induced labor, while both changes were more abrupt closer to delivery in spontaneous cases.

**Figure 6:**
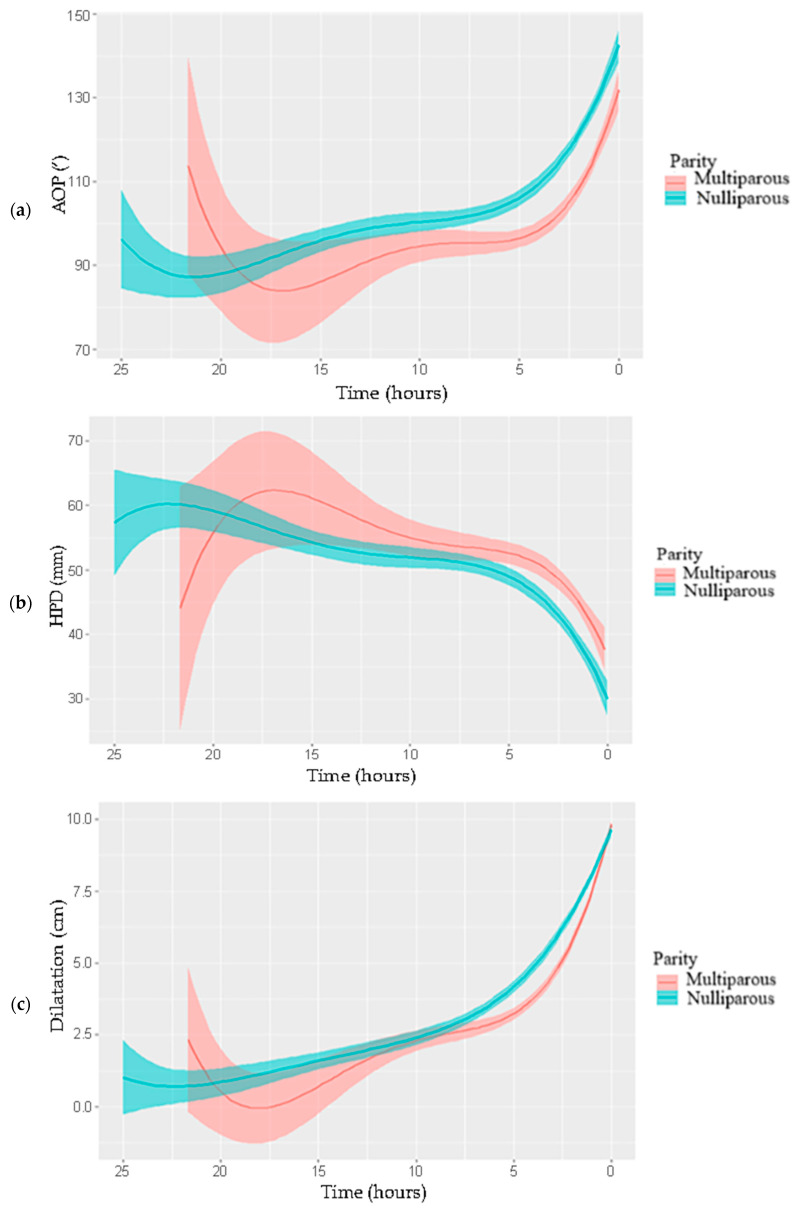
Changes in AoP (**a**), HPD (**b**), and dilatation (**c**) values until delivery by parity; multiparous vs. nulliparous.

The changes in AoP (a), HPD (b), and cervical dilatation (c) in parturients, based on parity (nulliparous vs. multiparous), are depicted in Figure 6. The trends of AoP, HPD, and dilatation over time were similar in both nulliparous and multiparous, with the difference being that the fluctuations were more pronounced in multiparous.

**Figure 7:**
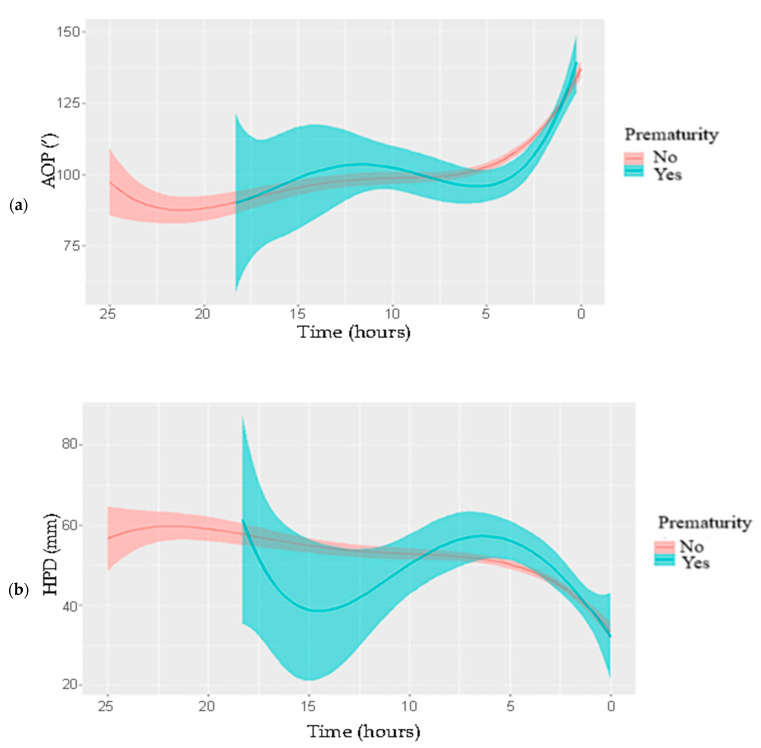

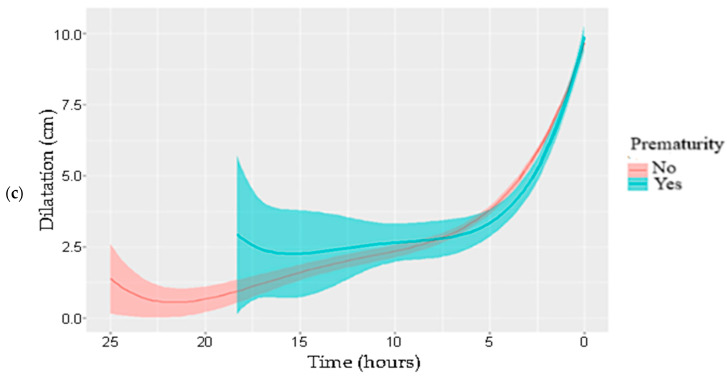
Changes in AoP (**a**), HPD (**b**), and dilatation (**c**) values until delivery associated by prematurity.

The changes in AoP (a), HPD (b), and cervical dilatation (c) in parturients, depending on whether the delivery was preterm or not are shown in Figure 7. In women who did not deliver preterm, AoP values showed a continuous increase, initially at a mild rate, which became steeper from five hours before delivery until birth. In contrast, in those who gave birth preterm, the values showed greater fluctuations, and the final increase also began five hours before delivery but was steeper compared to the other women. Similar findings were observed for the decrease in HPD values. However, cervical dilatation values followed a fairly similar pattern in both groups of women.

In Table 2, AoP, HPD, and dilatation values at the first and last examination of the women, by type of delivery are presented. Women’s first and last AoP, HPD, and dilatation measurements differed significantly by type of labor. More specifically, after Bonferroni correction, it was found that at the first examination, the AoP value was significantly lower in CS compared to OVD (92° vs. 105°, *p* = 0.007) and to VD cases (92° vs. 101°, *p* < 0.001). Also, at the first examination the HPD value was significantly higher in CS compared to OVD (55 mm vs. 50 mm, *p* < 0.001) and VD (55 vs. 49 mm, *p* < 0.001). At the first examination, the mean dilatation was significantly greater in VD compared to OVD cases (3 cm vs. 2 cm, *p* = 0.010) and CS (3 cm vs. 1 cm, *p* = 0.003). At the last examination, the AoP value and the dilatation was significantly lower in CS cases compared to OVD (104.5° vs. 118°, *p* < 0.001 and 6 cm vs. 10 cm, *p* < 0.001 respectively) and VD cases (104.5° vs. 114°, *p*= 0.009 and 6 cm vs. 10 cm, *p* < 0.001 respectively). Also, at the last examination, the HPD value was significantly higher in CS cases compared to OVD (49 mm vs. 43.5 mm, *p* = 0.009) and VD cases (49 mm vs. 43 mm, *p* = 0.003).

Via mixed linear models, it emerged that there was a negative correlation between AoP and HPD (β = −0.36, SE = 0.01, *p* < 0.001). Also, AoP values were positively correlated with participant dilatation (β = 4.12, SE = 0.20, *p* < 0.001), while HPD values were negatively correlated with participant dilatation (β = −1.90; SE = 0.15; *p* < 0.001). Through mixed linear models it was found that the relationship of dilatation with AoP and HPD is best described by a quadratic polynomial (both in the whole sample and by type of delivery), with the only exception being the correlation of dilatation and HPD in CS, where their relationship is best described by a first-degree polynomial (i.e., a linear relationship). AoP and HPD estimations during the active phase of labor via mixed linear models, in total sample and separately in OVD/VD and CS are presented in Table 3.

In the total population, at full dilation (10 cm), the mean AoP was 131.7° (95% CI: 129.2–134.3°) and the mean HPD was 37.2 mm (95% CI: 35.3–39.2 mm). In OVD/VD, at full dilation, the mean AoP was 133.4° (95% CI: 130.6–136.2°) and the mean HPD was 36.4 mm (95% CI: 34.3–38.5 mm). In CS cases, at full dilation, the mean AoP was 117.9° (95% CI: 111.6–124.2°) and the mean HPD was 45.1 mm (95% CI: 40.6–49.6 mm).

The predicted time to delivery according to various values of AoP, through mixed linear models, in OVD/VD cases, is presented in Table 4. When AoP is 78° the mean time to delivery was 9.01 h (95% CI: 8.50–9.51 h), when it is 110° the time is estimated at 5.05 h on average (95% CI: 4.71–5.39 h) and when it is 125° the time is estimated at 3.19 h on average (95% CI: 2.80–3.59 h). When the HPD is 60 mm the time to delivery is estimated to be 7.55 h on average (95% CI: 7.09–8.00 h), when it is 40 mm the time to delivery is estimated to be 3.92 h on average (95% CI: 3.53–4.30 h), and when it is 30 mm the time is estimated at 2.10 h on average (95% CI: 1.58–2.62 h).

## 4. Discussion

### 4.1. Main Findings

The active phase of labor (≥4 cm dilation) began approximately 5 h before delivery, accompanied by a sharp rise in AoP and a steep decrease in HPD. AoP increased in all delivery modes but at a slower rate in CS and OVD, particularly in CS. Cervical dilation progressed in all groups; continuously in OVD, more steeply in VD 5 h before delivery, and more gradually in CS. Epidural use was associated with steeper final changes in AoP, HPD, and dilation starting around 2.5 h before delivery, while oxytocin use led to similar but slightly earlier accelerations beginning 3–4 h before delivery. An AoP of 125° and an HPD of 40 mm corresponded to average delivery times of 3 and 4 h, respectively. CS cases had higher BMI and birthweight, while VD had shorter labor duration and lower induction rates. More pronounced changes in AoP, HPD, and dilation were observed in spontaneous labor compared to induced labor, and while patterns were similar in nulliparous and multiparous women, fluctuations were greater in the latter. Preterm births showed steeper and more variable changes in AoP and HPD, with cervical dilation patterns similar to full-term births.

### 4.2. Interpretation of the Findings

Visual comparisons indicate that the ultrasound descent pattern closely resembles the shape of the clinical curves published by Friedman [12]. According to Friedman’s curve, the descent started on average at 4 cm dilatation and the maximum rate of descent was observed at an average of 6 cm dilatation. According to the Hjartardottir’s ultrasonographic labor curves, rapid descent started at 7 to 8 cm of dilatation, however in both of the curves, delivery was observed approximately 4 h later [13]. The duration of the active phase of labor in Friedman’s curve was similar to the one observed in our study, approximately 5 h, whereas in Hjartardottir’s study, the active phase lasted longer (8.4 h). The onset of the active phase of labor was defined at 4 cm, according to the national guidelines. The observed difference may also result from changes in the obstetric population or clinical practices [2,14,15]. Additionally, approximately 5 h before delivery, the AoP values began to increase sharply and the HPD values to decrease sharply, which was similarly depicted in the Hjartardottir’s ultrasonographic labor curves [13]. In a study of women with induction of labor, those who delivered vaginally had steeper slopes of AoP and HPD against fetal head station and cervical dilation than those who had cesarean delivery [16].

Regarding the AoP, according to the study of Hjartardottir et al., in cases of CS, virtually no descent was observed, whereas a gradual descent was observed in OVD. Similarly, in our study, a lower rate of increase of AoP was observed in OVD and even lower in CS, compared to the continuous increase in VD [13]. As for the clinically assessed dilatation, a linear slope which was steepest for spontaneous deliveries and slightly less steep in the cases ending with OVD was noticed in the Hjartardottir’s study. A comparable slope that tapered off and plateaued at a mean of 8 cm dilatation, approximately 4 h prior to delivery was observed in cases that ended up in cesarean delivery [13]. In our study, dilatation increased regardless of the type of delivery, with the difference that in OVD the increase was continuous, in VD from 5 h onwards the increase was steeper and in CS it was less steep throughout the observation period.

As far as the use of epidural is concerned, studies have shown conflicting results of the impact of epidural analgesia on the progress of labor [17,18,19,20,21,22,23,24,25,26,27,28,29,30]. In our study, 36.2% of women received epidural. In Hjartardottir’s study, the curve of fetal head descent was slightly more sloping in women who received epidural. However, in the final stages before labor, the rate of increase of AoP and decrease of HPD were steeper, in cases of epidural. Similarly, in our study, the final ascent in AoP and dilatation, as well as the final descent in HPD, which started approximately 2.5 h before delivery, were steeper in cases who had an epidural, despite the fact that the curves were less steep in the initial phase of epidural administration.

Regarding the use of oxytocin, 65.8% of parturients received oxytocin and the final increase in AoP and dilatation, as well as the final decrease in HPD, that began 3–4 h before labor, were more accelerated in women who took oxytocin, which is similarly depicted in the patterns of Hjartardottir.

Finally, in this study, when the AoP was 125°, the mean time to delivery was 3.2 h and when the HPD was 40 mm, the mean time to delivery was 3.9 h. The ultrasonographic patterns from the study of Hjartardottir revealed the same predicted time to delivery when the AoP is 125°. However, when the HPD was 40 mm, the predicted time to delivery was 5.5 h compared to 3.9 h in our study. This could be explained by the different population; the higher BMI in the Greek pregnant population may explain the higher HPD measurements, which are significantly correlated with the higher BMI [31].

To our knowledge, labor curves distinguished based on parity (multiparous vs. nulliparous women), on type of onset of labor (induction of labor vs. spontaneous labor) and based on gestation age (preterm birth vs. term birth) have not been published before. The trends of AoP, HPD, and cervical dilatation values were similar in multiparous and nulliparous women, but more fluctuations were noticed in multiparous. The greater fluctuations in values in multiparous women are likely due to more compliant pelvic tissues, less resistance to descent, and faster, more variable engagement of the fetal head; rapid spikes in AoP during contractions, followed by slight reversals between contractions, resulting in a less smooth progression and curve. In women who experienced spontaneous labor onset, the rate of increase was more intense over a shorter time span, starting three to four hours before delivery. Similar findings were observed for the decrease in HPD values and the increase in cervical dilatation. That is reasonably explained because oxytocin was not used routinely in cases of induction of labor, as explained with detail. Regarding prematurity, the greater fluctuations and steeper changes in AoP and HPD in preterm births could be attributed to less stable fetal head engagement, higher mobility, and inconsistent pressure on the pelvic floor, while cervical dilation progresses more uniformly, as it follows a hormonally regulated, tissue-level process that becomes active once labor begins, regardless of gestational age.

Furthermore, the associations found in our study are in accordance with the literature. First, obesity is known to be strongly associated with increased odds of CS. Thus, a higher BMI increased the risk of CS after induction of labor in the groups with BMI 25–34.9 kg/m^2^ [32]. In another study, the overall mean BMI was higher among women who delivered via CS [33], which was in agreement with the findings of this study. According to the literature, longer active first stage of labor duration was associated with increased occurrence of OVD and CS, which was in accordance with this study [34]. Moreover, CS rates rose with increasing birthweight, in a total of 7528 deliveries [35], in agreement with our findings. Numerous studies in the literature compared women with induction of labor to women with spontaneous onset of labor and reported higher CS rates among women who underwent labor induction, in most cases nearly double the risk of CS, similarly to our study [36,37,38,39,40]. A multicenter cohort study revealed that nulliparous women had a two-fold risk of CS, which was in accordance with our findings [41].

### 4.3. Strengths and Limitations

The main strengths of this study were the prospective design and the large sample size. The inclusion of both multiparous and nulliparous women, inclusion of cases of induction of labor and preterm births, as well is considered another strength of this study. To our knowledge, this is the first study of determination of labor curves in the pregnant population of Greece. A potential limitation could be that the ultrasound examiners were not maternal-fetal medicine specialists. However, all of them received appropriate training and it has been shown that intrapartum sonographic skills can be easily obtained [42]. Furthermore, due to the small number of cases with persistent occiput posterior position at the last examination, labor curves could not be differentiated into occiput anterior vs. posterior position.

## 5. Conclusions

Objective, validated intrapartum ultrasound techniques were employed to characterize the pattern of fetal head descent during labor in a Greek population. To the best of our knowledge, this is the first study attempting to determine the labor curves based on ultrasonographic parameters in Greece. Further longitudinal studies may assist in prospectively validating our results in the other populations.

## Figures and Tables

**Table 1:** Maternal, pregnancy, and labor characteristics in the study population and by type of delivery.

			Type of Labor			*p*
*n* = 500		Total Sample	CS (*n* = 52; 10.4%)	OVD (*n* = 35; 7.0%)	VD (*n* = 413; 82.6%)	
*Maternal characteristics*						
	Age (years), mean (SD)	28.8 (6.5)	30 (5.4)	27.4 (6)	28.7 (6.7)	0.167 ‡
	ΒΜΙ, mean (SD)	30 (5.18)	32.03 (5.6)	29.94 (5.22)	29.75 (5.08)	0.011 ‡
*Pregnancy- and labor- related characteristics*						
	Gestational age (weeks), mean (SD)	38.8 (1.5)	38.9 (1.5)	39.1 (1.3)	38.8 (1.5)	0.384 ‡
	Preterm labor, *n* (%)	44 (8.8)	5 (9.6)	3 (8.6)	36 (8.7)	0.951 ++
	Nulliparous, *n* (%)	286 (57.2)	45 (86.5)	33 (94.3)	208 (50.4)	<0.001 +
	Epidural, *n* (%)	181 (36.2)	22 (42.3)	17 (48.6)	142 (34.4)	0.153 +
	Oxytocin, *n* (%)	329 (65.8)	40 (76.9)	27 (77.1)	262 (63.4)	0.050 +
	Labor onset, *n* (%)					
	Spontaneous	201 (40.2)	12 (23.1)	10 (28.6)	179 (43.3)	
	Induction	299 (59.8)	40 (76.9)	25 (71.4)	234 (56.7)	0.007 +
	Total labor duration, median (IQR)	8.3 (5–12)	15 (10–21)	12 (7–17)	8 (5–12)	<0.001 ‡‡
	Birth weight (gr), mean (SD)	3131.69 (471.23)	3267.88 (502.02)	3266.86 (380.56)	3103.09 (470.2)	0.012 ‡

+ Pearson’s chi-square test; ++ Fisher’s exact test; ‡ ANOVA; ‡‡ Kruskal–Wallis test.

**Table 2:** AoP, HPD, and dilatation values the first and last examination of the women, by type of delivery.

	Type of Labor			*p* Kruskal–Wallis Test	*p* ^1^ Pairwise Comparisons		
	CS	OVD	VD				
	Median (IQR)	Median (IQR)	Median (IQR)		CS vs. OVD	CS vs. VD	OVD vs. VD
At first examination							
AοP (°)	92 (84–102.5)	105 (89–113)	101 (92–112)	<0.001	*0.007*	<*0.001*	0.504
HPD (mm)	55 (51–61)	50 (44–53)	49 (42–55)	<0.001	<*0.001*	<*0.001*	0.917
Dilatation (cm)	1 (1–4)	2 (1–4)	3 (1–5)	<0.001	0.958	*0.003*	*0.010*
At last examination							
AοP (°)	104.5 (93–126)	118 (110–141)	114 (101–129)	0.002	<*0.001*	*0.009*	0.029
HPD (mm)	49 (40–56)	43.5 (29.5–49)	43 (35.5–51)	0.005	*0.009*	*0.003*	0.357
Dilatation (cm)	6 (3–10)	10 (10–10)	10 (10–10)	<0.001	<*0.001*	<*0.001*	0.763

Note. *p*-values in *italics* are significant after Bonferroni correction. ^1^ via Mann–Whitney test.

**Table 3:** AoP and HPD estimations during the active phase of labor via mixed linear models in total sample and separately in OVD/VD and CS deliveries.

	Dilatation	AοΡ (°)		HPD (mm)	
		Estimation	95% CI	Estimation	95% CI
Total sample	4 cm	102.6	101.1–104.0	50.1	49.0–51.1
	5 cm	105.6	104.0–107.2	48.6	47.3–49.8
	6 cm	109.4	107.7–111.2	46.8	45.4–48.1
	7 cm	113.9	112.0–115.8	44.8	43.3–46.2
	8 cm	119.1	117.1–121.1	42.5	40.9–44.0
	9 cm	125.1	122.9–127.3	40.0	38.3–41.7
	10 cm	131.7	129.2–134.3	37.2	35.3–39.2
OVD/VD	4 cm	103.4	102.0–104.9	49.5	48.4–50.6
	5 cm	106.6	104.9–108.3	48.1	46.8–49.4
	6 cm	110.5	108.6–112.5	46.4	44.9–47.8
	7 cm	115.1	113.1–117.2	44.3	42.8–45.9
	8 cm	120.5	118.3–122.7	42.0	40.3–43.7
	9 cm	126.6	124.2–129.0	39.4	37.5–41.2
	10 cm	133.4	130.6–136.2	36.4	34.3–38.5
CS	4 cm	95.4	91.3–99.4	54.1	51.8–56.4
	5 cm	97.1	92.7–101.5	52.6	50.2–55.0
	6 cm	99.6	94.9–104.3	51.1	48.4–53.8
	7 cm	103.0	98.1–107.9	49.6	46.6–52.6
	8 cm	107.1	102.0–112.3	48.1	44.6–51.5
	9 cm	112.1	106.5–117.6	46.6	42.6–50.5
	10 cm	117.9	111.6–124.2	45.1	40.6–49.6

Note. 95% CI: 95% Confidence Interval.

**Table 4:** Predicted time to delivery in OVD/VD cases, according to AoP and HPD values.

	Predicted Time to Delivery (Hours)	95% CI (Hours)
AoP (°)		
78	9.01	8.50–9.51
80	8.76	8.27–9.25
84	8.27	7.81–8.72
85	8.14	7.70–8.59
95	6.90	6.52–7.28
99	6.41	6.05–6.77
110	5.05	4.71–5.39
113	4.68	4.34–5.02
116	4.31	3.96–4.66
120	3.81	3.45–4.18
125	3.19	2.80–3.59
138	1.59	1.09–2.08
140	1.33	0.83–1.85
148	0.35	0.00–0.93
HPD (mm)		
60	7.55	7.09–8.00
54	6.46	6.08–6.84
50	5.73	5.38–6.09
48	5.37	5.02–5.72
40	3.92	3.53–4.30
38	3.55	3.15–3.96
37	3.37	2.96–3.79
36	3.19	2.77–3.62
35	3.01	2.57–3.45
30	2.10	1.58–2.62
20	0.29	0.00–0.99

## Data Availability

The original contributions presented in this study are included in the article. Further inquiries can be directed to the corresponding author.
